# Contemporary Management of Hodgkin Lymphoma and Chronic Lymphocytic Leukemia

**Published:** 2018-04-01

**Authors:** Sandra E. Kurtin, 1)The University of Arizona Cancer Center, Tucson, Arizona; 2)John Theurer Cancer Center, Hackensack, New Jersey McNeill

**Affiliations:** 1 The University of Arizona Cancer Center, Tucson, Arizona;; 2 John Theurer Cancer Center, Hackensack, New Jersey

## Abstract

Identifying key molecular attributes can help in the risk-adapted treatment of Hodgkin lymphoma and chronic lymphocytic leukemia. At JADPRO Live, presenters reviewed best practices for managing patients with the rapidly expanding armamentarium of treatments for these patients.

The identification of key molecular attributes has relevance in the risk-adapted treatment of Hodgkin lymphoma and chronic lymphocytic leukemia. Advanced practitioners in oncology play an important role in assessing patients and managing them with a rapidly expanding number of treatments, according to information presented at JADPRO Live 2017 by Sandra E. Kurtin, PhDc, AOCN®, ANP-C, of the University of Arizona Cancer Center, and Ann McNeill, RN, MSN, APN, of the John Theurer Cancer Center, Hackensack, New Jersey.

## HODGKIN LYMPHOMAS

As Ms. Kurtin reported, there are approximately 8,000 new cases of Hodgkin lymphoma (HL) per year, and in 2013 there were an estimated 193,545 people living with HL in the United States (Kamper-Jørgensen et al., 2013). Although a decrease in the number of deaths is a positive sign, older patients present a challenge. The most common presenting signs and symptoms include (Mauch et al., 1993) the following:

- Asymptomatic lymphadenopathy, most often in the neck of the mediastinum (60%–70%)
- Cough, retrosternal chest pain, or shortness of breath
- Pruritus (severe in many cases)
- Alcohol-induced pain in sites of disease
- B symptoms (common in stage III/IV disease): Fever, night sweats, and weight loss
- Eosinophilia
- Bone pain

Regarding diagnostic evaluation, Ms. Kurtin emphasized that HL is an inflammatory disease. This is evident morphologically and pathologically, with lymphocytes attracting dendritic cells, natural killer cells, eosinophils, and other inflammatory cells. "This is why needle biopsies in these patients are often nondiagnostic, showing just fibrosis, and excisional biopsies are recommended," she said.

Classical HL is the most common type of HL, but there are four subtypes based on differences in the appearance of the tumor cells and the composition of the microenvironment (Hoppe et al., 2017):

- Nodular sclerosis classical HL: disease above the diaphragm and mediastinal node involvement most common
- Mixed cellularity classical HL: liver involvement more common
- Lymphocyte-rich classical HL
- Lymphocyte-depleted classical HL: least common subtype; more likely to present with advanced stage disease, liver involvement, B symptoms

**Clinical Staging of Classical HL**

As Ms. Kurtin reported, staging and risk stratification, which has become the norm in hematologic malignancies, is complicated for HL, which is divided into "early-stage favorable," "early-stage unfavorable," and "advanced-stage disease" (Table 1). Unfavorable factors include bulky chest masses (> 10 cm or a mediastinal mass ratio > 0.33), extranodal involvement (at least three sites of disease), erythrocyte sedimentation rate (ESR) ≥ 50, and presence of B symptoms, including fever, night sweats, and weight loss (Ng & van Leeuwen, 2016; Table 1).

**Table 1:**
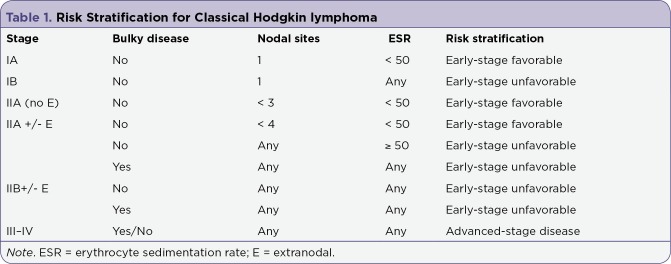
Risk Stratification for Classical Hodgkin lymphoma

**ABVD**

ABVD, a chemotherapy regimen used in the first-line treatment of HL, consists of concurrent treatment with doxorubicin (Adriamycin), bleomycin, vinblastine, and dacarbazine. Venous access can be an issue for these patients because doxorubicin and vinblastine are vesicants and dacarbazine is an extreme irritant (Canellos et al., 1992).

**Response-Adapted Frontline Therapy**

To maximize cures while minimizing late effects, to avoid undertreatment or overtreatment, reduce treatment-emergent adverse events, and reduce potential long-term effects, oncologists now utilize PET-adapted therapy, which relies on Deauville criteria and the PET 5-point scale (Barrington et al., 2014). "We don’t want to give an excess of a drug if we don’t have to, nor do we want to undertreat," she said. "Importantly, you want to have a Deauville score of 3 or less after two cycles."

According to Ms. Kurtin, positron emission tomography (PET) imaging at baseline and repeated after two cycles of treatment is standard to guide overall treatment and reduce the risks of treatment-related adverse events. "There are now patients with early-stage favorable disease who may only receive two cycles of ABVD, and if they’re PET-negative after two cycles, they’re not going to get any more chemotherapy," said Ms. Kurtin

Ms. Kurtin emphasized the importance of repeated pulmonary function testing and echocardiograms, particularly in older patients with Hodgkin lymphoma. "If we cure a patient, but he or she ends up with cardiopulmonary toxicities that are life-altering, we have compromised their quality of life. We want to be smart about the intensity of therapy," she said. 

"In some patients with compromised pulmonary function at baseline, leaving bleomycin out from the beginning may be advisable, or we can now substitute brentuximab vedotin for some patients," she added.

**Relapsed/Refractory Disease**

According to Ms. Kurtin, relapsed or refractory HL is a "whole different ball game. For younger patients, we are going to try to get them to transplant, but there are several options available to them," she said. "It’s important to consider the pattern of relapse, how long it’s been since their initial treatment, the type of initial response, and performance status."

A number of regimens are available for relapsed classical HL (Table 2). In addition, patients who relapse following autologous hematopoietic stem cell transplant (auto-HSCT) may be considered for an allogeneic stem cell transplant (allo-HSCT), and brentuximab vedotin may be used in maintenance therapy. Approved in 2011, brentuximab vedotin is an anti-CD30 antibody with the following indications for classical HL: after failure of auto-HSCT, in transplant-ineligible candidates after failure of at least two multi-agent chemotherapy regimens; and in patients at high risk of relapse or progression as post auto-HSCT (Table 2).

**Table 2:**
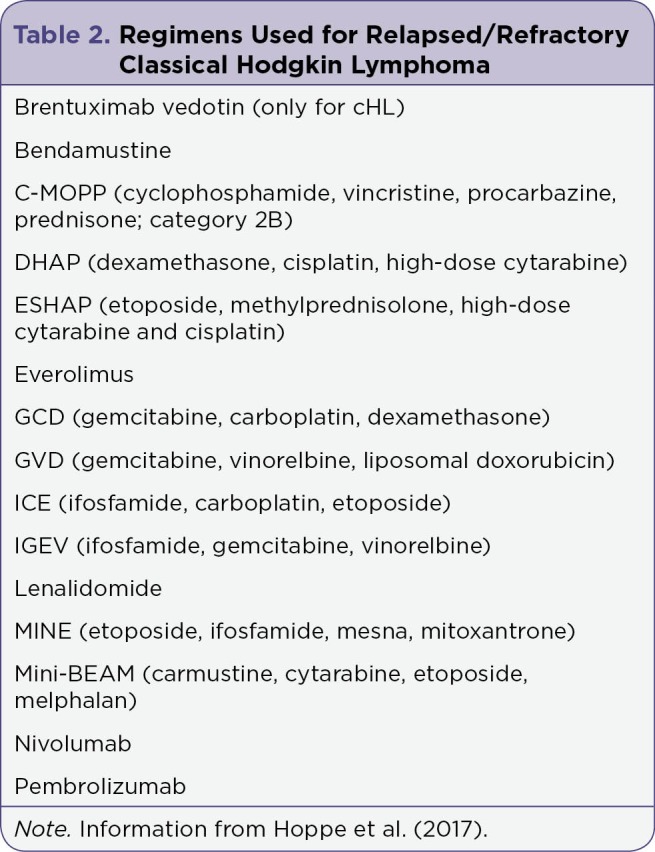
Regimens Used for Relapsed/Refractory Classical Hodgkin Lymphoma

"Brentuximab vedotin is dosed every 3 weeks and is given over 30 minutes, so it’s really not too hard on the patients in terms of scheduling," said Ms. Kurtin, who also added that neuropathy, however, is something that needs to be monitored closely in these patients.

**Novel Agents**

Several novel agents have been recently approved, and numerous trials are now exploring potential therapeutic targets. Checkpoint inhibitors (nivolumab [Opdivo] and pembrolizumab [Keytruda]) are currently available for relapsed or progressive disease following a transplant and post-transplant maintenance therapy. In the KEYNOTE-087 trial, at a median follow-up of 9.4 months, the overall response rate was 69% for pembrolizumab (Moskowitz et al., 2016).

"Even in patients with high-risk disease or those who are heavily pretreated, there were a good number of responses," said Ms. Kurtin, who noted, however, that there are adverse events associated with immune checkpoint inhibition. "Augmented immune response driven by T-cell activation creates the potential for autoimmune-related inflammation of normal tissues, and onset is often delayed compared to standard therapies" (Michot et al., 2016).

## CHRONIC LYMPHOCYTIC LEUKEMIA

As Ms. McNeill reported, with approximately 18,900 new cases diagnosed per year, chronic lymphocytic leukemia (CLL) is a rare non-Hodgkin lymphoma (National Cancer Institute, 2017). With a median age of diagnosis of 71 years, it is also a disease of the elderly (especially white males). The good news, said Ms. McNeill, is that the 5-year overall survival rate has increased tremendously recently, going from 67% to nearly 82%.

There are two staging systems for CLL: the Rai staging system, which is common in the United States, and Binet staging, which is more commonly used in Europe. In addition to staging, genomic alterations are information (Table 3). The presence of a del(17p)/TP53 mutation, serum β2 greater than 3.5 mg/dL, immunoglobulin heavy-chain variable region gene (IgHV) mutation status, and patient age are used as prognostic factors to determine overall survival (Parikh & Shanafelt, 2016). Although the presence of a 17p deletion is probably the most significant, unfavorable prognostic variable for CLL, said Ms. McNeill, there are other genomic alterations with clinical value, including 13q deletion (unfavorable) and 11q deletion (favorable). Unmutated IgHV is also unfavorable. The presence or absence of these alterations affects median time to progression and median survival (Cramer & Hallek, 2011). The presence of elevated white blood cell count alone, however, is not a significant adverse prognostic factor (Silverman, Franssen, Buckstein, & Imrie, 2002).

**Table 3:**
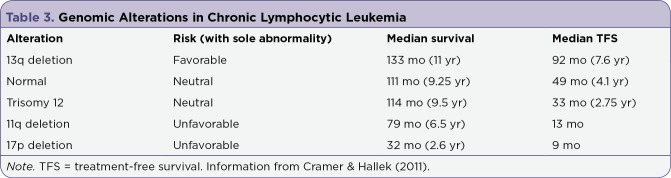
Genomic Alterations in Chronic Lymphocytic Leukemia

"All of us who have treated patients with CLL have patients with very, very high white counts and lymphocyte counts in the hundreds of thousands, and these patients are living happily, for a very long time," said Ms. McNeill.

Indications for treatment include the extent and severity of disease manifestations. These include significant B symptoms (night sweats, fever without infection, severe fatigue, and unintentional weight loss), generalized pruritus, tumor burden, bone marrow failure, and immune dysfunction (Hallek, 2015).

**FDA-Approved Drugs to Treat Chronic Lymphocytic Leukemia**

As Ms. McNeill reported, many drugs are approved to treat CLL. Fludarabine, approved since 1991, remains a preferred regimen for younger and fit patients and works well in combination with rituximab, with response rates of 80% in previously untreated patients. Nevertheless, providers should be aware of hematologic and infectious toxicities.

"It’s very important that there is a long-term depletion of CD-positive T lymphocytes in this population, so we are going to be very aware of how we are affecting the bone marrow with the use of this agent," she said.

Ibrutinib (Imbruvica) is an oral gentamicin used for CLL based on the results of two studies. In the RESONATE trial, ibrutinib was compared with ofatumumab in previously treated patients with CLL and small lymphocytic lymphoma (SLL), and in the RESONATE-2 trial, ibrutinib was compared with chlorambucil in treatment-naive CLL/SLL patients (Burger et al., 2015; Byrd et al., 2014). A significant improvement in overall response rates, progression-free survival, and overall survival was seen in both studies in the ibrutinib arms. Blood analysis of CLL patients treated with ibrutinib, however, has shown that lymphocytosis is common (Woyach et al., 2014).

"Lymphocytosis usually occurs within the first month after ibrutinib dosing, but it resolves relatively quickly, usually within 8 months," said Ms. McNeill. "Some patients still have lymphocytosis after a year, but this persistent lymphocytosis in the absence of any other symptom does not represent a clonal evolution or progression of disease, and progression-free survival is not inferior for these patients who do experience this prolonged lymphocytosis."

In addition, said Ms. McNeill, providers should be aware of fatigue, cytopenias, diarrhea, musculoskeletal pain, and atrial fibrillation. Patients with cardiac risk factors, acute infections, and a history of atrial fibrillation should be monitored closely for atrial fibrillation and atrial flutter; when symptomatic, discontinuation of ibrutinib should be considered. Finally, ibrutinib studies have also shown a higher incidence of bleeding events, although the mechanism is not clearly understood.

**Clonal Evolution Relapsed/Refractory Chronic Lymphocytic Leukemia**

According to Ms. McNeill, clonal evolution, the acquisition of new cytogenetic abnormalities during the disease course, is very important for advanced practitioners to be aware of.

"It is critical for us to know that the disease at the initial presentation and initial diagnosis can be very different at relapse. Actually, mutations are acquired throughout a patient’s journey," she explained. "For example, it is critical to test for deletion 17p or 11q, high-risk abnormalities that are associated with inferior overall survival."

Acquisition of low or intermediate abnormalities, such as trisomy 12, deletion 13q, and IgH translocation, have shown no difference in overall survival (Woyach et al., 2014).

For the second-line treatment of CLL, fit patients can be treated with ibrutinib, idelalisib (Zydelig), venetoclax (Venclexta), chemoimmunotherapy, and allogeneic hematopoietic stem cell transplant. In addition, clinical trials are always an option (Hallek, 2015).

"If there is progression after 2 years," said Ms. McNeill, "the standard of care is repeating first-line therapy, although with all the novel agents currently available, most practitioners would probably go to one of the newer agents like lenalidomide (Revlimid)."

When compared to rituximab and placebo, idelalisib, a PI3 kinase inhibitor and an oral agent, has also shown significant improvements in progression-free survival, overall response rate, and overall survival (Furman et al., 2014). Approved in 2016 for relapsed CLL with deletion 17p, venetoclax, a BCL2 inhibitor, is one of the newer agents and is associated with higher overall response rates in relapsed patients, in phase I and II trials (Souers et al., 2013).

"What’s really important to note about venetoclax is that we can see very rapid tumor lysis syndrome (TLS), and for that reason, there is a risk-based TLS prophylaxis," she said. "If we can manage early debulking and the risk of TLS, however, venetoclax is very well tolerated."

Ofatumumab (Arzerra), a CD20 monoclonal antibody, was approved in 2009 for patients with CLL refractory to fludarabine and alemtuzumab (Campath). Although well tolerated, Ms. McNeill advised close monitoring, especially at first infusion. Providers also need to be aware of neutropenia. Another anti-CD20 monoclonal antibody, obinutuzumab in combination with chlorambucil has shown improved overall response rates in previously untreated patients with CLL, when compared with rituximab/chlorambucil or chlorambucil alone. Again, said Ms. McNeill, advanced practitioners should look out for infusion-related reactions as well as cytopenias and infections, such as pneumonia (Goede et al., 2014).
